# Apoptosis and Pro-inflammatory Cytokine Response of Mast Cells Induced by Influenza A Viruses

**DOI:** 10.1371/journal.pone.0100109

**Published:** 2014-06-12

**Authors:** Bo Liu, Di Meng, Tangting Wei, Siyi Zhang, Yanxin Hu, Ming Wang

**Affiliations:** 1 Key Laboratory of Zoonosis of Ministry of Agriculture, College of Veterinary Medicine, China Agricultural University, Beijing, China; 2 Zhongmu Institutes of China Animal Husbandry Group, Beijing, China; Innsbruck Medical University, Austria

## Abstract

The pathogenesis of the influenza A virus has been investigated heavily, and both the inflammatory response and apoptosis have been found to have a definitive role in this process. The results of studies performed by the present and other groups have indicated that mast cells may play a role in the severity of the disease. To further investigate cellular responses to influenza A virus infection, apoptosis and inflammatory response were studied in mouse mastocytoma cell line P815. This is the first study to demonstrate that H1N1 (A/WSN/33), H5N1 (A/Chicken/Henan/1/04), and H7N2 (A/Chicken/Hebei/2/02) influenza viruses can induce mast cell apoptosis. They were found to do this mainly through the mitochondria/cytochrome c-mediated intrinsic pathway, and the activation of caspase 8-mediated extrinsic pathway was here found to be weak. Two pro-apoptotic Bcl-2 homology domain 3 (BH3) -only molecules Bim and Puma appeared to be involved in the apoptotic pathways. When virus-induced apoptosis was inhibited in P815 cells using pan-caspase (Z-VAD-fmk) and caspase-9 (Z-LEHD-fmk) inhibitors, the replication of these three subtypes of viruses was suppressed and the secretions of pro-inflammatory cytokines and chemokines, including IL-6, IL-18, TNF-α, and MCP-1, decreased. The results of this study may further understanding of the role of mast cells in host defense and pathogenesis of influenza virus. They may also facilitate the development of novel therapeutic aids against influenza virus infection.

## Introduction

Influenza A virus (IAV) is one of the most common respiratory pathogens. It is notorious for its unique potential to cause global pandemics and epidemics in animals and humans of all age groups. It has considerable morbidity and high fatality rates. Several studies suggest that fatal lung tissue injury triggered by the cytokines dysregulation (called “cytokine storm”), which is produced by excessive immune inflammation response, makes a critical contribution to the mortality of influenza [1]–[5].

Mast cells are enriched at tissue sites that interface closely with the external environment and so a crucial sentinel role in host defense against pathogens, such as bacteria, parasites, and viruses [6]–[8]. The role of mast cells during influenza virus infection has been ignored until recently. Data from this and other research groups have demonstrated the involvement of mast cells in IAV infection [9]–[11]. One recent study by the present team has shown that very large numbers and very high levels of pro-inflammatory cytokines and chemokines are produced and secreted in P815 mast cells during IAV infection (unpublished data). This indicates that mast cells may contribute to the pathogenesis of IAV infection.

Apoptosis, or programmed cell death, is a genetically controlled process distinct from necrosis. It is characterized by chromatin condensation, DNA fragmentation, membrane blebbing, cell shrinkage, and finally the formation of apoptotic bodies. It occurs in many pathological processes, such as cancer and microbial infection [12]–[14]. The mechanisms underlying apoptosis are highly complex. So far, two primary pathways have been described, the extrinsic, or death receptor, pathway which involves upstream activation of caspase 8, and the intrinsic, or mitochondrial, pathway which involves upstream activation of caspase 9. Both these pathways converge at downstream activation of caspase 3 or/and 7 [13]. IAV has been shown to induce apoptosis in a variety of cell types both *in vitro* and *in vivo* [15]–[17]. The present study was performed to determine whether IAV infection could induce apoptosis in mast cells and to identify the pathways involved in the process.

The role of apoptosis induced by IAV infection has been hotly debated. Influenza-virus-induced apoptosis is considered one of the most effective host defense mechanisms against invading viruses. However, the virus has mechanisms by which it can utilize apoptosis to facilitate its own replication and spread [18], [19]. Apoptosis may also be involved in the symptoms of IAV infection, including lung tissue injury during IAV infection [19], [20]. In this way, virus-induced hyper-production of cytokines during inflammatory response and apoptosis have a definitive role in the pathogenesis of influenza and are intrinsically linked [20]. Defining the mechanisms of apoptosis and the pro-inflammatory response may facilitate the development of novel therapeutic aids against influenza virus infection.

The molecular mechanisms underlying apoptosis and the pro-inflammatory cytokine response in mast cells infected with influenza viruses were examined here. These results are the first to demonstrate that H1N1 (A/WSN/33), H5N1 (A/Chicken/Henan/1/04), and H7N2 (A/Chicken/Hebei/2/02) influenza viruses can induce mast cell apoptosis. They do so mainly through the mitochondria/cytochrome c-mediated intrinsic pathway. Activation of caspase 8-mediated extrinsic pathway was found to be weak. Two pro-apoptotic BH3-only molecules Bim and Puma appeared to be involved in the apoptotic pathways. When virus-induced apoptosis was inhibited in P815 cells by pan-caspase (Z-VAD-fmk) and caspase-9 (Z-LEHD-fmk) inhibitors, the replications of the three subtypes of virus were suppressed and the secretions of pro-inflammatory cytokines and chemokines, including IL-6, IL-18, TNF-α, and MCP-1, were decreased. The results of the present study may further our understanding of the role of mast cells in host defense and pathogenesis of influenza virus and may facilitate the development of novel therapeutic aids against influenza virus infection.

## Materials and Methods

### Viruses and Cell Cultures

The avian influenza viruses H5N1 (A/Chicken/Henan/1/04) [9] and H7N2 (A/Chicken/Hebei/2/02) used in this study were isolated from infected chicken flocks and propagated in the allantoic cavities of 10-day-old embryonated chicken eggs for 24 to 48 h at 37°C. The human influenza virus H1N1 (A/WSN/33) was provided by Dr. George F. Gao of the Institute of Microbiology, CAS, China, and the working stocks were generated in MDCK cells. All experimentations with H5N1 viruses were conducted in a biosafety level 3 (BSL-3) containment laboratory approved by the Ministry of Agriculture of China.

Mouse mastocytoma cell line P815 and Madin-Darby canine kidney cell line MDCK were provided by the Cell Resource Center of Peking Union Medical College (Beijing, China). Cells were cultured in Dulbecco’s modified Eagle’s medium (DMEM) (HyClone Laboratories, UT, U.S.) containing 10% fetal bovine serum (HyClone Laboratories), 100 U/ml penicillin, and 100 µg/ml streptomycin at 37°C with 5% CO_2_.

### Viral Infection

Cells were seeded onto 6-well (1×10^6^/well) or 12-well (5×10^5^/well) plates and cultured for 24 h to allow monolayer formation. Before infection, viruses were diluted with DMEM to equal titers, as determined by standard plaque assays as described below. Monolayers were then washed three times with DMEM and infected with viruses at a multiplicity of infection (MOI) of 0.1 for 1 h at 37°C. They were gently shaken every 10 min. After washing, DMEM supplemented with 1% bovine serum albumin was added to each well and allowed to incubate for the indicated periods at 37°C.

### Hemagglutination (HA) Assay

The supernatants of mock or virus-infected cell cultures were harvested at the indicated points in time, and HA assays were performed to quantify influenza A virus protein. Viral titers were assessed by the ability to agglutinate red blood cells, as described previously [21]. The assay was carried out in V-bottom microtiter plates. Fifty microliters supernatant samples were serially diluted two-fold using PBS and mixed with an equal volume of 0.5% (v/v) chicken erythrocytes, then incubated for 20–60 min at room temperature. Positive wells formed an adherent, homogeneous layer of erythrocytes, and negative reactions appeared as dots at the bottom of the plates. Viral titers were determined by the highest supernatant dilution that could cause erythrocytes to agglutinate completely. Titers were expressed in hemagglutinating units (HAU).

### Real-time Quantitative PCR

Total RNA was extracted from cells homogenized in Trizol reagents (Invitrogen, Carlsbad, U.S.), and 0.2 µg DNase I-treated RNA was reverse transcribed into cDNA using universal primers for IAV (Uni 12) with an EasyScript First-Strand cDNA Synthesis Super Mix (TransGen Biotech, China) according to the manufacturer’s instructions [22]. Reactions were performed in triplicate using a Power SYBR Green PCR Master Mix (Applied Biosystems, Warrington, U.K.) and run on an Applied Biosystems 7500 system. The mRNA expression levels of viral NS1 gene (forward primer, 5′-GCA ATT GGA ATC CTC ATC GG-3′; reverse primer, 5′-CAA CTC GTT TCG CCA TGT AGC-3′) were determined using the relative quantification method, which was normalized to β-actin (forward primer, 5′-GAG ACC TTC AAC ACC CCG C-3′; reverse primer, 5′-ATG TCA CGC ACG ATT TCC C-3′), served as an internal standard. These mRNA levels were compared to those of cells 3 h post infection and quantified using the 2^−ΔΔCT^ method. The amplifications were performed as follows: a 10 min hot start at 95°C followed by 40 cycles of denaturation at 95°C for 15 s, annealing at 55°C for 30 s, and extension at 72°C for 40 s.

### Plaque Assay

Plaque assays were performed to determine viral concentration as described previously [9]. Briefly, MDCK monolayer cells at >90% confluence in 6-well plates were washed with DMEM and infected with 10-fold serially diluted virus inoculum. After 1 h of incubation at 37°C, the inoculum was removed and washed. Then cell monolayers were overlaid with semisolid agar containing 0.5 µg/ml trypsin tosylsulfonyl phenylalanyl chloromethyl ketone (TPCK) (Sigma-Aldrich). Plaques were fully developed after 60–72 h at 37°C and 5% CO_2_. They were then fixed and stained with 1% crystal violet. The plaques were counted and the concentration of the initial viral suspension in PFU (plaque-forming unit)/ml was calculated.

### Transmission Electron Microscopy (TEM)

P815 cells were mock-treated or infected with viruses for 12 h, then trypsinized and fixed using 2.5% (v/v) glutaraldehyde in PBS for 2 h at 4°C. After the primary fixation, the samples were rinsed with PBS, then post-fixed in 1% osmium tetroxide, washed, and dehydrated in series of ethanol solutions. The dehydrated pellets were embedded in epoxy resin, and 70-nm sections were cut. Then the sections were placed on copper sieves and stained with uranyl acetate and lead citrate. Images of the sections were observed under a JEM-1230 TEM (Jeol, Japan Electronics Co., Ltd. Tokyo, Japan).

### TUNEL Assay

Apoptotic cells were examined using terminal deoxynucleotidyl transferase-mediated dUTP-biotin nick end labeling (TUNEL) assay, which was performed using an In Situ Cell Death Detection Kit in accordance with the manufacturer’s instructions (Roche, Philadelphia, PA, U.S.). Briefly, P815 cells were seeded onto polylysine-coated slides in 12 wells (5×10^5^/well) plates. Then, 12 h after infection, cells were rinsed twice with PBS, fixed on the polylysine-coated slide with 4% formaldehyde for 15 min, and permeabilized with 0.5% Triton X-100 in PBS for 15 min. Cells were rinsed twice with PBS. TUNEL reaction mixture was added to the slides and incubated at 37°C in a humidified atmosphere in the dark for 60 min. The slides were rinsed three times in PBS for 5 min each. At last, all slides were stained with 3 µg/ml 4′, 6′ -diamidine-2-phenylindole (DAPI) (Sigma-Aldrich) for 5 min to visualize nuclei. After another three washes, the slides were examined under a laser scanning confocal microscope (Leica TCS SP5 II, Leica Microsystems, Wetzlar, Germany).

### Flow Cytometric Analysis of Apoptosis

The apoptotic responses of P815 cells were examined at the indicated times after infection using an Annexin V-FITC Apoptosis Detection Kit (eBioscience, San Diego, CA, U.S.) according to the manufacturer’s instructions. Flow cytometric analysis was performed on a BD FACSCalibur using Cell Questpro software (BD Biosciences). Taxol (Sigma-Aldrich, Beijing, China). A concentration of 25 nM served as a positive inducer of apoptosis [23]. Cells were incubated at 37°C for the specified periods.

### Western Blot

Cells were harvested at the indicated times after infection, washed with PBS, and lysed with RIPA lysis buffer containing protease inhibitor cocktail (Beyotime Institute of Biotechnology, Beijing, China). Protein concentrations were determined using a BCA protein assay kit (Beyotime Institute of Biotechnology). Equal amounts of protein were separated on 12% SDS-PAGE gel and transferred to a polyvinylidene difluoride (PVDF) membrane (Millipore, Beijing, China). The membranes were blocked using 5% non-fat dry milk (BD Biosciences) at room temperature for 2 h, washed, and probed using the specified antibodies. Caspase 3 (8G10) rabbit mAb, caspase 8 polyclonal antibody (mouse-specific), cleaved caspase-8 (Asp387) polyclonal antibody (mouse-specific), caspase 9 polyclonal antibody (mouse-specific), cleaved caspase-9 (Asp353) polyclonal antibody (mouse-specific), Apaf-1 (D5C3) rabbit mAb, Cyt C (136F3) rabbit mAb, PARP (46D11) rabbit mAb, Bcl-2 (50E3) rabbit mAb, Bcl-xL (54H6) rabbit mAb, Bim (C34C5) rabbit mAb, Puma polyclonal antibody (rodent-specific), α-Tubulin (11H10) rabbit mAb, COX IV (3E11) rabbit mAb, and corresponding horseradish-peroxide-conjugated secondary antibody were obtained from Cell Signaling Technologies (Danvers, MA, U.S.). Protein bands were visualized using Western Lightning Plus-ECL (Perkin Elmer, MA, U.S.). β-actin served as a loading control.

### Subcellular Fractionation Extraction

To test the release of cytochrome C from the mitochondria, subcellular fractionation extraction was performed using the MS861 Cell Fractionation Kit (Mitosciences) following the manufacturer’s instructions. Then the expression levels of cytochrome C in cytoplasmic and mitochondria fractions was analyzed by Western blot.

### Inhibition of Apoptosis Using Specific Inhibitions

P815 cell monolayers were pre-incubated with pan-caspase (Z-VAD-fmk), caspase-8 (Z-IETD-fmk), and caspase-9 (Z-LEHD-fmk) inhibitor (all from R&D Systems, Minneapolis, MN, U.S.) at a concentration of 100 µM (diluted with DMSO) for 12 h. They were then mock-treated or infected with virus as described above. The same concentration of inhibitors was immediately added after 1 h of viral incubation. Samples were collected 24 h after infection.

### Cytokine and Chemokine Quantification

The IL-1β, IL-6, IL-18, IFN-γ, TNF-α, TGF-β, and MCP-1 concentrations in the supernatants of mock- and virus-infected cell cultures were determined using ELISA kits (eBioscience) according to the manufacturer’s instructions.

### Statistical Analysis

Statistical analysis was performed using two-way ANOVA and the SPSS software suite (version 12.0), and a *P* value of <0.05 was considered statistically significant. Results are expressed as mean ± standard deviation (SD) of at least three independent experiments.

## Results

### Influenza a Viral Infection Induced Apoptosis of P815 Mast Cells

**Figure 1:**
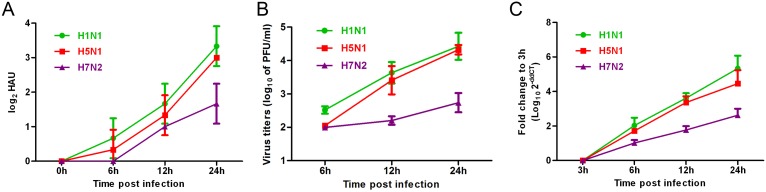
Mast cells supported productive replication of influenza A viruses. P815 cells were mock-treated or infected with the three subtypes of influenza viruses at an equal MOI of 0.1 for the periods specified. Culture supernatants were collected, and total viral protein quantification and viral titers were determined using a (A) hemagglutination (HA) assay and (B) standard plaque assay. (C) Cells were homogenized in Trizol and relative viral NS1 gene quantification was determined using real time PCR. The results shown here were pooled from three independent replicates.

To determine whether IAV infection can induce apoptosis in mast cells, the replication kinetics of H1N1, H5N1, and H7N2 were examined in the P815 mast cell line. As shown in Figure 1, all three subtypes of IAVs replicated productively in P815 cells as measured by HA assay, plaque formation, and viral NS1 gene expression. The replications of H1N1 and H5N1 were more efficient than those of H7N2, indicating that IAVs replicate well in mast cells with some degree of tropism selectivity.

**Figure 2:**
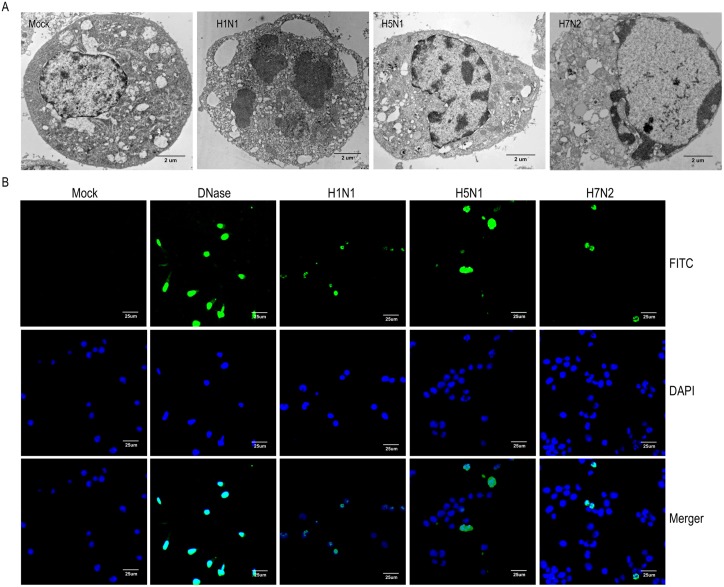
Apoptosis was induced in influenza A viruses infected P815 cells. P815 cells were mock-treated or infected with H1N1, H5N1, or H7N2 at an MOI of 0.1 for 12 h. (A) Transmission electron microscopy showed ultrastructure of mock-treated and virus-infected cells. (B) A TUNEL assay was used to measure apoptosis in P815 cells using DNase as a positive control. Blue indicates nuclear staining and green indicates positive TUNEL staining due to the presence of apoptosis. The results shown here are representative of three different donors.

During the process of IAV replication, extensive cytopathic effects (CPEs) were observed in P815 cells. Visualized transmission electron microscopy was used to determine whether cell apoptosis was taking place induced by IAV replication in P815 cells. As shown in Figure 2A, typical apoptotic morphological alterations were observed in the nuclei of P815 cells 12 h after IAV infection. In H1N1-infected cells, large apoptotic bodies moved from the nucleus to the cytoplasm (Figure 2A), which indicated that the cells underwent apoptosis during a late phase. Apoptotic responses were similar in H5N1- and H7N2-infected cells. They were characterized by chromatin condensation and aggregation at the periphery of the nucleons, plasma membrane continuity, and cell body shrinkage (Figure 2A). In contrast, intact cytoplasmic membranes, clear cytoplasm, intact nuclear membranes, and normal nucleoli were observed in mock-treated cells (Figure 2A). These data suggest that IAV infection may cause apoptosis in P815 cells. To confirm the occurrence of apoptotic responses, in situ TUNEL, an assay that detects DNA strand breakage, which is a hallmark of apoptosis, was performed. Positive signals of apoptosis were detected in all the three subtypes of IAVs infected P815 cells but not in mock-treated cells. DNaseI served as a positive control (Figure 2B).

**Figure 3:**
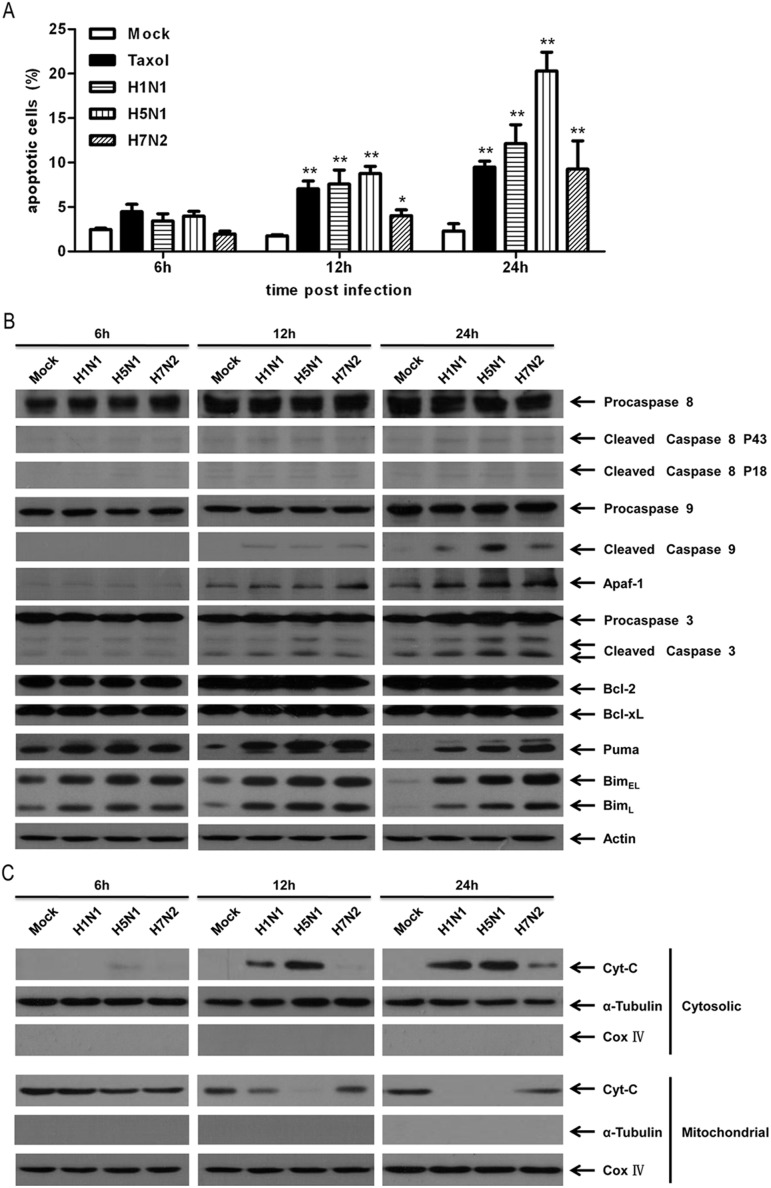
Apoptosis of P815 cells was further measured at specified times after influenza A virus infection. P815 cells were treated or infected as described in Figure 1 and harvested at 6 h, 12 h, and 24 h post-infection. (A) Quantification of the total number of apoptotic cells (early and late-stage apoptosis) by flow cytometric analysis with Taxol as a positive control. Asterisks indicate statistically significantly higher values than among mock-treated cells (*P<0.05 and **P<0.01). (B) Immunoblotting analysis of the expression levels of apoptosis-associated proteins, caspase 3, caspase 8, caspase 9, Apaf-1, Bcl-2, and Bcl-xL, with β-actin as a loading control. (C) Immunoblotting analysis of the cytosolic and mitochondrial Cyt C expression after fractionation. α-Tubulin and cytochrome oxidase subunit IV (Cox IV) were used as specific markers for the cytosolic and mitochondrial fractionations, respectively. Shown here are representative results of three separate experiments.

Next, the relative prevalence of apoptosis was quantified in IAV-infected P815 cells at specific times after infection. FITC-conjugated Annexin V and propidium iodide (PI) double staining were followed by cytometric analysis. Taxol at a concentration of 25 nmol/L served as the positive control. As shown in Figure 3A, apoptosis (early combination with late apoptosis) was marginally detectable at 6 h after infection but subsequently became dramatically higher than in mock-treated cells during the infection process. Moreover, the overall (early and late phases together) proportion of apoptosis in H5N1-infected cells was the highest among the three subtypes of IAVs at all the detected points in time, dropping to approximately 4% at 6 h after infection (*P*<0.05), and increasing to more than 20% at 24 h after infection (*P*<0.01), about twice as high as H7N2, which showed the least ability to induce apoptosis (Figure 3A). In addition, the expression levels of zymogen and active cleaved fragments of executioner caspase 3 were determined (Figure 3B). The results were exactly the same as those of flow cytometric analysis.

Taken together, the results given above suggest that all the three subtypes of IAVs, H1N1, H5N1, and H7N2, can induce apoptotic responses in P815 mast cells in a time-dependent manner. H5N1 induced the greatest amount of apoptosis among all the three subtypes.

### Influenza a Viruses Induced Apoptosis in P815 Cells Mainly via the Classical Intrinsic Caspase-9 Pathway

To further explore the molecular mechanism of apoptosis triggered in IAV-infected P815 cells, substrate cleavage and activation of extrinsic apoptotic marker caspase 8 and intrinsic apoptotic marker caspase 9 were subjected to Western blot analysis. The degradation of pro-caspase 8 in IAVs infected cells was weak at all times after infection and was only a little stronger than in mock-treated control cells (Figure 3B). The activated cleaved fragments of caspase 9 were detectable at 12 h after infection and significantly higher at 24 h after infection in IAV-infected cells than in mock-treated cells (Figure 3B). This strongly suggested that mitochondria-mediated intrinsic apoptosis was mainly involved in IAV induced apoptosis in mast cells. This was further verified by the dramatically release of cytochrome C (cyt C) from the mitochondria and up-regulated expression of Apaf-1 of IAV infected cells (Figure 3B and 3C). Both these proteins are essential to the activation of pro-caspase 9 and formation of apoptosomes.

The expression levels of Bcl-2, Bcl-xL, Puma and Bim, members of Bcl-2 family, were also measured. The expression levels of Bcl-2 and Bcl-xL, which inhibited apoptosis by binding to the pro-apoptotic members of the Bcl-2 family, did not appear to be affected by IAV infection (Figure 3B) [24]. However, the expression levels of pro-apoptotic BH3 -only molecules Bim and Puma were significantly up-regulated at 12 h and 24 h post-infection in IAV-infected cells than in mock-treated cells (Figure 3B). These results suggested that Bim and Puma might play an important role in mast cells apoptosis upon IAV infections.

**Figure 4:**
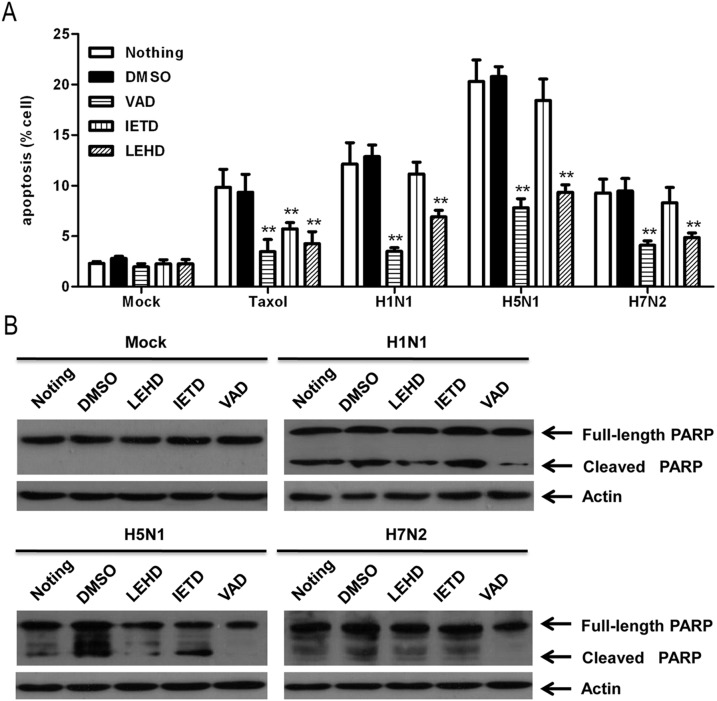
Caspase inhibitors suppressed the apoptosis in P815 cells infected with influenza A virus. VAD (abbreviation of Z-VAD-fmk, pan-caspase inhibitor), IETD (abbreviation of Z-IETD-fmk, caspase-8 inhibitor), and LEHD (abbreviation of Z-LEHD-fmk, caspase-9 inhibitor) were added before and after infection with influenza A viruses, and P815 cells were harvested 24 h after infection. (A) Apoptosis was quantified using flow cytometric analysis. Asterisks indicate that the inhibitor-incubated groups were statistically significantly different from the DMSO-incubated groups, as indicated by ANOVA. (B) The expression of full-length and cleaved PARP in P815 cells was measured using Western blot analysis. Graphs show results of one representative case (out of three).

In order to confirm the identity of the pathway involved in IAV-induced apoptosis of mast cells, caspase inhibition experiments were performed as described in the materials and methods section. As shown in Figure 4A and as detected by flow cytometric analysis, the VAD (pan-caspase inhibitor) had the most pronounced effect, reducing apoptosis by about 9%, 13%, and 5% in H1N1-, H5N1-, and H7N2-infected cells, respectively (*P*<0.01). The effect of LEHD (caspase-9 inhibitor) was a little weaker than that of VAD (pan-caspase inhibitor), but the rates of apoptosis were still significantly lower than among mock-treated or DMSO incubated controls (Figure 4A). The IETD (caspase-8 inhibitor) had little effect (Figure 4A). The inhibition of apoptosis was investigated by measuring the activation of caspase-activated poly (ADP-ribose) polymerase (PARP) using Western blot analysis. PARP is a substrate of multiple executioner caspases and the activated cleavage of this protein serves as a marker of cells undergoing apoptosis [13]. After VAD (pan-caspase inhibitor) and LEHD (caspase-9 inhibitor) inhibition, the levels of the cleaved PARP fragments in H1N1-, H5N1-, and H7N2-infected mast cells were significantly lower than in mock-treated and DMSO groups, and the effects of IETD (caspase-8 inhibitor) treatments were markedly weaker than those of other treatments (Figure 4B).

Collectively, these results indicated that the classical intrinsic apoptotic pathway was activated in all the three subtypes of IAV infection.

### Caspase Inhibitors Decreased the Viral Replication and the Release of Pro-inflammatory Cytokines and Chemokines

**Figure 5:**
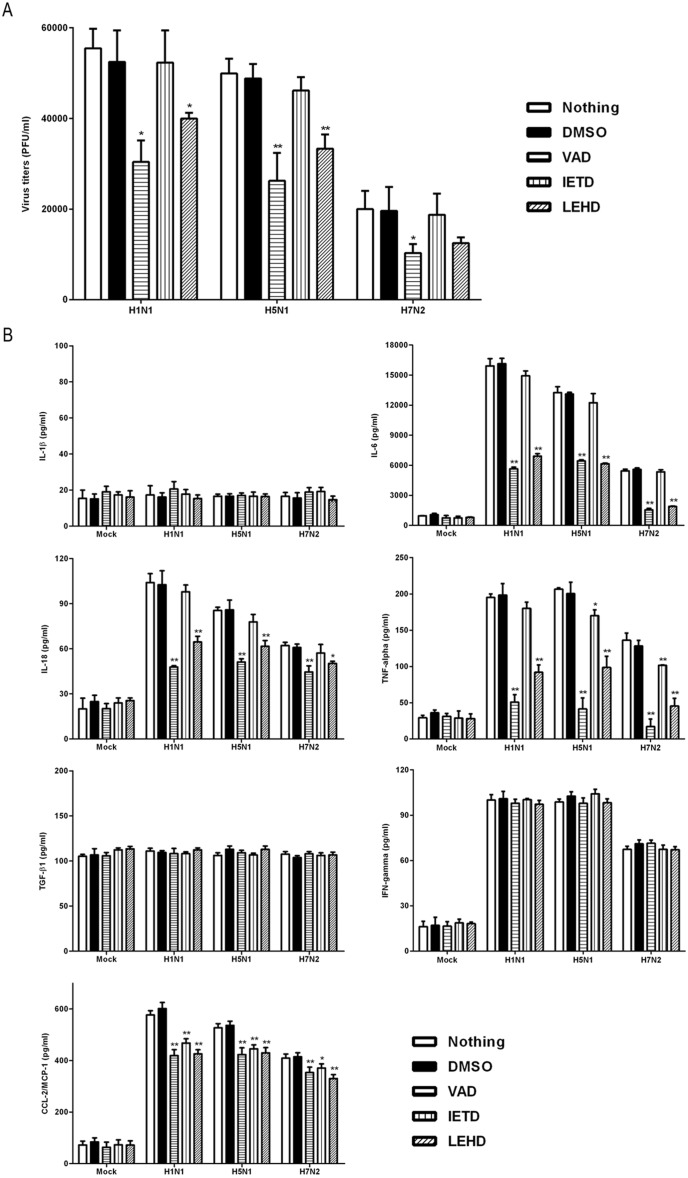
Inhibition of apoptosis suppressed the viral replication and release of pro-inflammatory cytokines and chemokines. P815 cells were treated as described in Figure 4. Cell supernatants were harvested 24 h after infection. (A) Viral titers were determined using a standard plaque assay. Results shown were pooled from three independent repeats. (B) Expression levels of IL-1β, IL-6, IL-18, TNF-α, TGF-β1, IFN-γ, and MCP-1 were analyzed using ELISA. Statistically significant differences between the inhibitor-treated groups and corresponding DMSO controls are indicated by * (*P*<0.05) and ** (*P*<0.01). Results shown are pooled from three independent repeats. ND, not detectable.

Because apoptosis and the pro-inflammatory response both have a definitive role in the pathogenesis of influenza virus, a preliminary attempt was here made to identify the intrinsic relationships between apoptosis and viral replication and the pro-informatory response. Treatment of IAV-infected P815 cells with VAD (pan-caspase inhibitor) and LEHD (caspase-9 inhibitor) dramatically decreased viral titers 24 h after infection, and IETD (caspase-8 inhibitor) did not (Figure 5A). In this way, apoptosis appears to be beneficial to IAV replication. This conflicts with the hypothesis that virus-induced apoptosis has an antiviral role.

In addition, the effects of the three inhibitors on the release of pro-inflammatory cytokines and chemokines, including IL-1β, IL-6, IL-18, IFN-γ, TNF-α, TGF-β, and MCP-1, were examined. The expression of IL-1β and TGF-β was barely detectable and had no significant differences across the experimental groups (Figure 5B). This suggested that the H1N1, H5N1, and H7N2 viruses used in this study did not induce the expression of these two cytokines. All three subtypes of viruses induced increased expression of IL-6, IL-18, IFN-γ, TNF-α, and MCP-1, particularly IL-6 and MCP-1 (Figure 5B). The levels of all detectable cytokines and chemokines were considerably higher in H1N1- and H5N1-infected cells than in H7N2-infected cells 24 h after infection (Figure 5B). Treatment with the VAD (pan-caspase inhibitor) rendered the release of IL-6, IL-18, TNF-α, and CCL-2 significantly lower than in IAV-infected cells alone and in DMSO controls (*P*<0.01), LEHD (caspase-9 inhibitor) did the same (*P*<0.01). IETD (caspase-8 inhibitor) had a less pronounced suppressive effect of on the secretions of CCL-2 and TNF-α than VAD (pan-caspase inhibitor) and LEHD (caspase-9 inhibitor) did (Figure 5B). None of these three inhibitors affected the secretion of IFN-γ during IAV infection. IAV-induced apoptosis was found to promote the secretion of some specific kinds of pro-inflammatory cytokines and chemokines in infected mast cells.

## Discussion

Mast cells are crucial to optimal immune response during infection, including bacterial, parasitic, and viral infection. However, the role of mast cells in influenza A virus infection is currently unknown, especially in apoptosis and inflammatory response. The present study provides the first evidence that the H1N1 (A/WSN/33), H5N1 (A/Chicken/Henan/1/04), and H7N2 (A/Chicken/Hebei/2/02) viruses can induce apoptosis in mast cells. These data also showed that this apoptosis takes place mainly through the mitochondria-mediated intrinsic pathway. Results also showed that inhibition of apoptosis suppressed of viral replications and the secretion of specific kinds of pro-inflammatory cytokines and chemokines.

Induction of apoptosis is multifactorial and complicated. Different IAV strains differ in their ability to induce apoptosis. This phenomenon is also specific to cell type. Here, three different influenza virus subtypes (H1N1, H5N1, and H7N2) were used to confirm the induction of apoptotic response in P815 mast cells through several apoptosis detection assays, including the observations of typical morphological features using transmission electron microscopy, positive TUNEL staining, flow cytometry quantitative analysis, and molecular evidence concerning the activation of executioner caspase 3. This is the first work to demonstrate the induction of apoptosis by IAV infection in mast cells, although Anderson and colleagues have already shown that mast cells can undergo apoptosis triggered by dengue viral infection but not by dsRNA analog poly (I:C), a synthetic mimic of influenza virus [25].

The death-receptor-mediated extrinsic pathway can be induced by influenza viral infection. This has been extensively reported and the relevant mechanisms are well established [26]–[29]. However, little is known about the involvement of the mitochondria/cytochrome c-mediated intrinsic apoptotic pathway in IAV-induced apoptosis. Recently, avian H9N2 has been shown to induce apoptosis through both extrinsic and intrinsic pathways in human tracheobronchial epithelial cells [30]. However, the present results showed the intrinsic apoptotic cascade to be clearly activated in P815 mast cells infected with IAVs, as indicated by 1) activation of caspase 9; 2) release of Cyt C from the mitochondria and increased expression of Apaf-1; and 3) the action of caspase-9 inhibitor (Z-LEHD-fmk), which inhibited virus-induced apoptosis very effectively. However, the extrinsic pathway appeared not to participate in mast cell apoptosis as induced by IAV, as demonstrated by the feeble cleavage and activation of zymogen pro-caspase 8, although TNF-α was significantly induced during the infection. The reason why the existence of TNF-α did not activate the extrinsic pathway may be that TNF-α was induced only during a late stage of infection (24 h after infection). The same results, that the extrinsic pathway was not activated, were observed in the apoptosis of PK-15 cells infected with swine influenza virus [31]. This is unlike H5N1, in which H5N1-encoded NS1 protein can induce apoptosis via caspase 8 but not via caspase 9 activation in MDCK and human lung epithelial cells [32], [33]. Several apoptosis signaling pathways that are induced by influenza virus infection have been described in the past decade. This phenomenon appears to be specific to cell type and viral components. For example, the PB1-F2 protein encoded by the influenza virus seems to target the mitochondria specifically and cause apoptosis [34], [35]. Viral matrix protein M1 has been found to be indirectly involved in mitochondria-mediated activation of caspase [36]. However, whether PB1-F2 and M1 play decisive roles in the intrinsic pathway of P815 cells merits further study.

The BH3-only proteins (Bad, Bid, Bim, Bmf, Bik, Hrk, Noxa and Puma), members of the Bcl-2 protein family, have a conserved BH3 domain that can bind to the anti-apoptotic Bcl-2 proteins to promote apoptosis [37]. In addition to this, it was demonstrated that the BH3-only proteins Bim and Puma played an important role in regulating mast cell apoptosis [38], [39]. Consistent with the previous reports, the present study showed that Bim and Puma might be involved in mast cells apoptosis upon IAV infections, though the related mechanisms need further study.

Apoptosis is a host defense mechanism. It limits viral replication. The influenza virus is believed to overcome this defense by undergoing efficient multiplication before apoptosis is complete [20], [40]. IAV production was here found to be impaired when apoptosis was inhibited using pan-caspase or caspase 9 inhibitors in P815 mast cells, suggesting that apoptosis may play a role in release of the virus. Several previous studies have also shown that the induction of apoptosis is essential to propagation of the influenza virus [41]–[43].

Apoptosis, unlike necrosis, is a non-inflammatory event. Apoptotic cells are phagocytized before they release their contents. This general conclusion has been questioned in IAV-infected human macrophages, produce IL-1 and IL-18 by provoking caspase 1 and caspase 3 when they undergo apoptosis [43]–[45]. Here, the production levels of several pro-inflammatory cytokines and chemokines (IL-6, IL-18, IFN-γ, TNF-α, and MCP-1/CCL-2) in IAV-infected mast cells were elevated despite apoptosis. Inhibition of caspase activation and hence of apoptosis caused a decrease in the release of all of these cytokines and chemokines except IFN-γ. However, it was previously reported that caspase inhibitors could increase the expression of pro-inflammatory cytokines in epithelial cells during IAV infection [46]. The reason for this difference is probably due to the lower replication rates of the viruses after inhibition of caspase activation reported in the present work, while the previous study by Brydon *et al* shown that viral titers were not affected by the caspase inhibitors. However, whether these low levels of cytokine and chemokine secretion are attributable to lower viral replication capacity, but further experiments are required to confirm this because not all the cytokines showed reduced concentrations. In this scenario, IFN-γ is a special case.

Collectively, the results of the present study suggest that virus-induced hyper-production of pro-inflammatory cytokines and chemokines (cytokine storm) and apoptosis are intrinsically linked in mast cells during IAV infection. H5N1 viral RNA was detected in the lungs and tracheas, where apoptosis and inflammation were both prominent, but little viral RNA was found in the livers, where apoptosis and inflammation were not observed [47]. Mice infected with the highly virulent 1918 H1N1 showed marked and sustained activation of pro-inflammatory and apoptotic pathways, while those infected with less virulent viruses saw a less pronounced host immune response accompanied by less severe pathology [48]. These *in vivo* findings suggest that apoptosis plays an important role in the mediation of inflammation and subsequent tissue damage in severe pathogenesis. As such, the present *in vitro* study of the cellular responses of mast cells to influenza A virus infection may provide novel insight and facilitate the development of therapeutic aids suitable for use against influenza virus infection.
